# Epileptic–Dyskinetic Encephalopathy Associated with a *PPP3CA* Variant: Expansion of the Phenotypic Spectrum

**DOI:** 10.1002/mdc3.70635

**Published:** 2026-04-09

**Authors:** Bruno Antunes Contrucci, Ursula Thomé, Larissa Aparecida Batista, Ana Laura Volpi Martins Lorenzoni, Carla Andrea Cardoso Tanuri Caldas, Americo Ceiki Sakamoto, Israel Gomy, Lisandra Mesquita Batista, Marcela Lopes de Almeida, Ana Paula Andrade Hamad, Guillermo Andrey Ariza Traslaviña

**Affiliations:** ^1^ Department of Neurology Ribeirão Preto Medical School, University of São Paulo Ribeirão Preto Brazil; ^2^ Department of Medical Genetics Ribeirão Preto Medical School, University of São Paulo Ribeirão Preto Brazil

**Keywords:** developmental and epileptic encephalopathy, movement disorders, *PPP3CA gene*

Epileptic–dyskinetic encephalopathies are a subgroup of developmental and epileptic encephalopathies (DEE/EDE), defined by the coexistence of early‐onset epilepsy and hyperkinetic movement disorders leading to significant neurodevelopmental impairment.^1^, ^2^ Previous reports of *PPP3CA*‐related disorders mainly describe severe developmental delay and early‐onset developmental and epileptic encephalopathy with refractory seizures, while movement disorders have not been systematically characterized, suggesting an important gap in current genotype–phenotype correlations.^3^, ^4^, ^5^, ^6^, ^7^, ^8^, ^9^

Male born to healthy, unrelated parents after an uncomplicated 41‐week caesarean delivery, the child exhibited early developmental delay characterized by hypotonia and limited social interaction.

At 6 months of age, the patient developed epileptic spasms and was diagnosed with Infantile Epileptic Spasm Syndrome. Resolution of hypsarrhythmia required high‐dose corticotropin after failure of high‐dose prednisolone and vigabatrin. Despite multiple antiseizure medications, seizure control remained suboptimal, progressing to daily polymorphic seizures, including tonic and myoclonic episodes and abnormal eye movements with autonomic features. Serial EEGs showed multifocal epileptic encephalopathy.

In parallel, a movement disorder emerged at approximately 2 months of age and persisted throughout the first year, initially characterized by distal dystonia affecting the lower limbs, with progressive extension to the upper limbs and involvement of both hands and feet. After the first year, chorea became evident, with prominent oromandibular dyskinesias and subsequent involvement of the shoulder and pelvic girdles, resulting in a diffuse, persistent choreiform pattern (Fig. 1).

**Figure 1:**
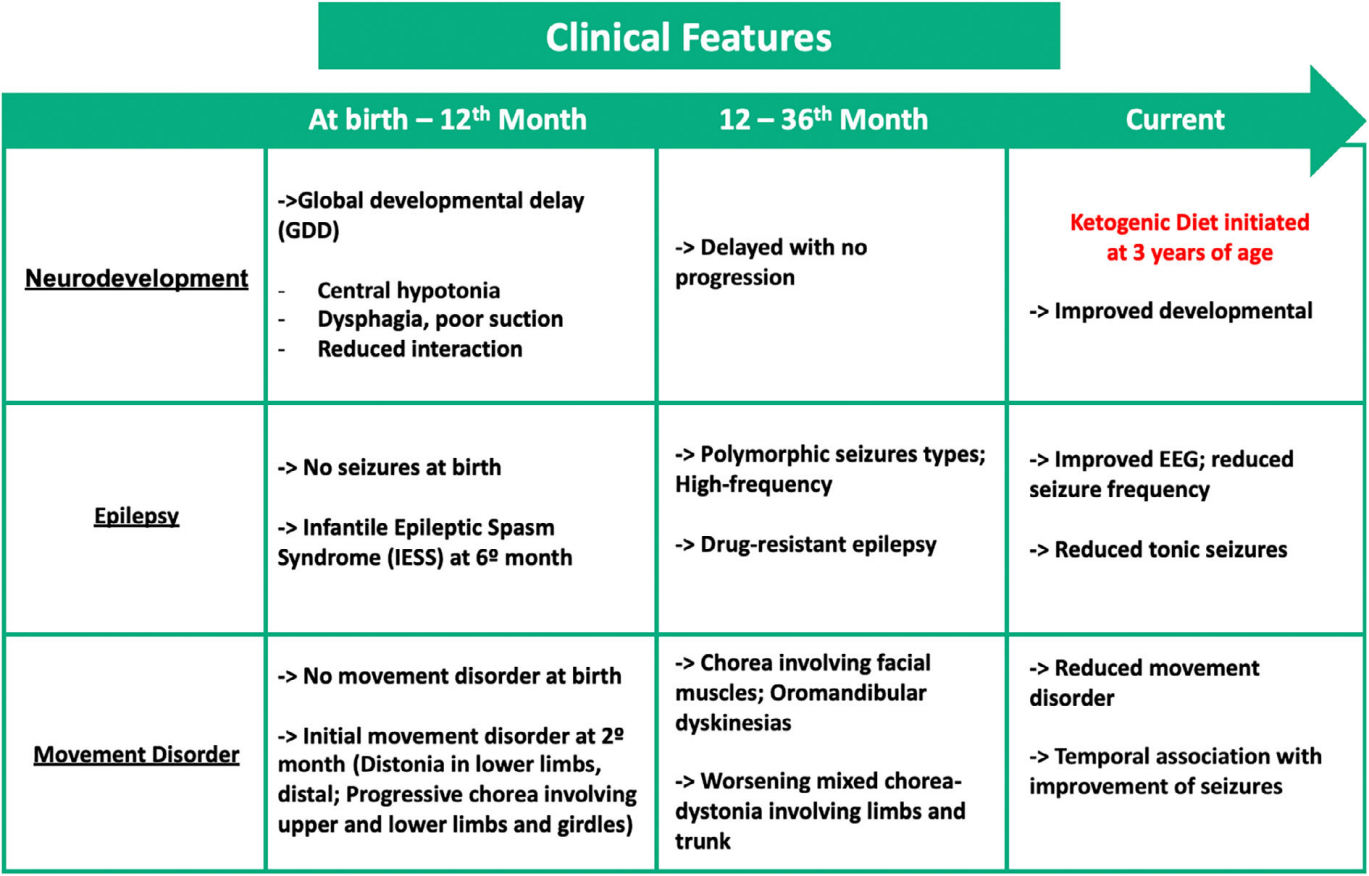
Chronological clinical evolution, summarizing developmental features, epilepsy and movement disorder.

At 3 years of age, examination revealed axial and appendicular hypotonia and a severe hyperkinetic movement disorder affecting the face, trunk, and limbs (Video 1). Movements were purposeless and fluctuating. Functional interaction was minimal, with a mixed chorea–dystonia pattern involving proximal and distal segments and prominent orofacial dyskinesias.

**Video 1:** Three‐year‐old patient with axial and appendicular hypotonia, accompanied by a marked hyperkinetic movement disorder affecting the face, trunk, and limbs.

Exacerbations occurred during intercurrent infections and sleep–wake cycle disruptions, usually in association with seizure worsening. Trials of haloperidol, biperiden, clonazepam, and gabapentin failed to provide sustained benefit.

Brain magnetic resonance imaging (MRI) demonstrated diffuse cerebral atrophy with preservation of the basal ganglia and normal MR spectroscopy. Metabolic evaluation was unremarkable. No involvement of other organ systems was identified, and ophthalmologic and audiologic assessments were normal. Whole‐exome sequencing and mitochondrial DNA analysis by next‐generation sequencing revealed a heterozygous variant in *PPP3CA* (NM_000944.5:c.701A>G; p.Asp234Gly). Parental testing confirmed the variant as de novo.

At the most recent follow‐up, the patient exhibited profound global developmental delay, multifocal epileptic encephalopathy, and a persistent hyperkinetic movement disorder. Antiseizure and symptomatic treatments were optimized. A ketogenic diet was introduced as adjunctive therapy to improve epileptic encephalopathy. Given the patient's age and risk of adverse effects, the diet was initiated at a 2:1 ratio with regular monitoring of ketone levels and blood glucose.

After 4 months of ketogenic diet therapy, with good tolerability and sustained ketosis, a clinically perceptible reduction in seizure frequency was observed, accompanied by improved EEG background organization and reduced epileptiform discharges. This was temporally associated with attenuation of the hyperkinetic movement disorder, predominantly choreiform. Given the short follow‐up and concomitant treatment optimization, this association should not be interpreted as causal.

This case expands the phenotypic spectrum associated with *PPP3CA*, particularly regarding the coexistence of early‐onset developmental and epileptic encephalopathy and a prominent hyperkinetic movement disorder. *PPP3CA* encodes the catalytic subunit of calcineurin, a protein involved in intracellular signaling, regulation of endocytosis, and synaptic vesicle transmission.^3^ Depending on the functional impact of the variant (gain‐ or loss‐of‐function), a spectrum of neurodevelopmental disorders may occur.^4^ Loss‐of‐function variants typically result in early‐onset developmental and epileptic encephalopathy (IECEE1; OMIM #617711).^3^, ^4^ Although movement disorders have occasionally been mentioned in association with *PPP3CA*‐related phenotypes, their clinical relevance and semiological characterization remain poorly defined.

The variant identified in our patient (NM_000944.5:c.701A>G; p.Asp234Gly) is located in an evolutionarily conserved region and is absent from population databases, including gnomAD,^10^ meeting a pathogenicity criterion with a moderate level of evidence (PM2).^5^ Its de novo occurrence in a patient with unaffected parents provides strong evidence of pathogenicity (PS2).^5^ In silico analyses using multiple predictive tools consistently indicate a deleterious effect on the protein, supporting pathogenicity at a supporting level of evidence (PP3).^5^ In addition, a distinct missense substitution affecting the same codon has previously been reported as pathogenic (Variation ID: 1452512), providing moderate evidence of pathogenicity (PM5).^5^ Furthermore, the variant is located within a critical functional domain of *PPP3CA*, a gene known to be highly constrained for missense variation. Based on ACMG criteria,^5^ considering one strong, two moderate, and one supporting lines of evidence for pathogenicity, with no evidence supporting benignity, the variant was classified as likely pathogenic.

Previous reports of *PPP3CA* loss‐of‐function variants describe global neurodevelopmental delay and refractory seizures with heterogeneous semiology.^6^, ^7^ However, a consistent association with hyperkinetic movement disorders has not been clearly characterized. Although such disorders have been mentioned, detailed semiological characterization and prevalence data are lacking, likely reflecting phenotypic heterogeneity and underrecognition in earlier cohorts.^8^, ^9^ To date, no reports have described ketogenic diet use targeting either epilepsy or movement disorders in PPP3CA‐related disease.

The increasing recognition of genetically determined epilepsies associated with movement disorders underscores the ongoing need to delineate novel genotype–phenotype correlations.^11^ Advancing this understanding has direct implications for clinical management and may broaden therapeutic possibilities, as illustrated by this case, in which meaningful clinical improvement was observed following ketogenic diet initiation.

## Author Roles

(1) Research project: A. Conception, B. Organization, C. Execution; (2) Statistical analysis: A. Design, B. Execution, C. Review and critique; (3) Manuscript: A. Writing of the first draft, B. Review and critique.

B.A.C.: 1A, 1C, 2A, 2B, 3A.

U.T.: 1B, 2C, 3A.

L.A.B.: 1B, 1C.

A.L.V.M.L.: 1B, 2C, 3A.

C.A.C.T.C.: 1C, 3A.

A.C.S.: 2C, 3B.

I.G.: 2C, 3B.

L.M.B.: 1C; 2B.

M.L.A.: 3A, 3B.

A.P.A.H.: 2C, 3B.

G.A.A.T.: 2B, 2C, 3B.

## Disclosures

**Ethical Compliance Statement:** We confirm that we have read the Journal's position on issues related to ethical publication and affirm that this report is consistent with those guidelines. All procedures performed involving human participants were conducted in accordance with the ethical standards of the institutional and/or national research committee, and with the 1964 Declaration of Helsinki and its later amendments. Written informed consent was obtained from the patient's legal guardians for participation and for publication of clinical data, images, and video material.

**Funding Sources and Conflicts of Interest:** No specific funding was received for this work. The authors declare that there are no conflicts of interest relevant to this article.

**Financial Disclosures for the Previous 12 Months:** None.

## Data Availability

The data that supports the findings of this study are available in the supplementary material of this article.
